# Role of Corneal Stromal Cells on Epithelial Cell Function during Wound Healing

**DOI:** 10.3390/ijms19020464

**Published:** 2018-02-04

**Authors:** Bhavani S. Kowtharapu, Radovan Murín, Anselm G. M. Jünemann, Oliver Stachs

**Affiliations:** 1 Department of Ophthalmology, Rostock University Medical Center, 18057 Rostock, Germany; anselm.juenemann@med.uni-rostock.de (A.G.M.J.); oliver.stachs@med.uni-rostock.de (O.S.); 2 Department of Medical Biochemistry, Jessenius Faculty of Medicine in Martin, Comenius University in Bratislava, Malá hora 4D, 03601 Martin, Slovakia; murin@jfmed.uniba.sk

**Keywords:** cornea, stromal fibroblasts, epithelial cells, antibody microarray, EMT-like changes

## Abstract

Following injury, corneal stromal keratocytes transform into repair-phenotype of activated stromal fibroblasts (SFs) and participate in wound repair. Simultaneously, ongoing bi-directional communications between corneal stromal-epithelial cells also play a vital role in mediating the process of wound healing. Factors produced by stromal cells are known to induce proliferation, differentiation, and motility of corneal epithelial cells, which are also subsequently the main processes that occur during wound healing. In this context, the present study aims to investigate the effect of SFs conditioned medium (SFCM) on corneal epithelial cell function along with substance P (SP). Antibody microarrays were employed to profile differentially expressed cell surface markers and cytokines in the presence of SFCM and SP. Antibody microarray data revealed enhanced expression of the ITGB1 in corneal epithelial cells following stimulation with SP whereas SFCM induced abundant expression of IL-8, ITGB1, PD1L1, PECA1, IL-15, BDNF, ICAM1, CD8A, CD44 and NTF4. All these proteins have either direct or indirect roles in epithelial cell growth, movement and adhesion related signaling cascades during tissue regeneration. We also observed activation of MAPK signaling pathway along with increased expression of focal adhesion kinase (FAK), paxillin, vimentin, β-catenin and vasodilator-stimulated phosphoprotein (VASP) phosphorylation. Additionally, epithelial-to-mesenchymal transition (EMT) regulating transcription factors Slug and ZEB1 expression were enhanced in the presence of SFCM. SP enriched the expression of integrin subunits α4, α5, αV, β1 and β3 whereas SFCM increased α4, α5, αV, β1 and β5 integrin subunits. We also observed increased expression of Serpin E1 following SP and SFCM treatment. Wound healing scratch assay revealed enhanced migration of epithelial cells following the addition of SFCM. Taken together, we conclude that SFCM-mediated sustained activation of ZEB1, Slug in combination with upregulated migration-associated integrins and ERK (Extracellular signal-regulated kinase)-FAK-paxillin axis, may lead to induce type 2 EMT-like changes during corneal epithelial wound healing.

## 1. Introduction

Corneal stromal keratocytes are neural crest-derived, quiescent, mesenchymal cells of the stroma and are capable of transforming into repair-phenotype of activated stromal fibroblasts (SFs) following injury [1]. Bi-directional communication between epithelial-stromal cells plays a crucial part in the event of corneal tissue repair [2] in which epithelial cells were stimulated to proliferate by mitogenic factors produced by SFs [3]. Stromal keratocytes produce hepatocyte and keratinocyte growth factors that are in turn induce proliferation, differentiation, and motility of corneal epithelial cells by paracrine mechanisms, which are also subsequently the main processes that occur during corneal tissue repair [3,4,5]. During the course of wound healing, cultured stromal keratocytes acquire characteristics similar to those of in vivo activated myofibroblasts [6]. Additionally, conditioned media (CM) collected from SFs (SFCM) has been shown to contain epidermal, basic fibroblast growth factors and contribute to the activation of corneal epithelial cells [3,7,8]. Furthermore, activated SFs also contribute to nerve regeneration following injury. Proteomic analysis of the SFCM from activated SFs reported more than 130 proteins which are predicted to regulate focal adhesion, nerve and tissue regeneration [9].

Sensory neuropeptide substance P (SP) plays an indispensable role during wound recovery phase by functioning as an injury-inducible messenger [10,11]. SP is expressed in the corneal epithelial cells and keratocytes along with its neurokinin receptor 1 [12] and is well known to participate in the mitigation of epithelial and stromal lesions [13,14,15]. SP is also one of the several proteins that are present in the SFCM [9]. Increased SP levels were observed in cultured trigeminal neurons after treatment with corneal epithelial conditioned media [16] and also in keratocytes following injury [13]. SP enhances motility of different cell types [13,17,18] and is known to activate mitogen-activated protein kinases (MAPKs), phosphoinositide 3-kinase-Akt, protein kinase C and EGFR signaling pathways [14,19].

Epithelial-to-mesenchymal transition (EMT) is a biological process that allows polarized, stationary epithelial cells to adopt a mesenchymal cell phenotype with increased migratory capacity through a series of biochemical changes. The EMT that occurs during the repair-associated events of tissue regeneration has been classified as type 2 EMT [20,21] and is also involved in the corneal healing process [22,23]. Integrins, by interacting with extracellular matrix (ECM) components, can facilitate interactions between cells and also transduce signals that influence cell shape, proliferation, adhesion, stress fiber formation and motility [24,25]. Furthermore, they are also known to involve in a variety of inside-out and outside-in signaling events during the process of EMT [26,27,28] and corneal repair mechanisms [29,30,31,32].

The purpose of the present study was to study the effect of activated SFs on corneal epithelial function in the context of wound repair. Primary SFs were cultured and the collected SFCM was added to corneal epithelial cells in culture. Simultaneously, SP was also used to study its effect on corneal epithelial cells. Antibody microarrays were employed to profile differentially expressed cell surface markers and cytokines in the corneal epithelial cells after stimulation with SFCM and SP. Furthermore, signaling studies were performed on corneal epithelial cells with emphasis to identify the roles of SP and SFCM in mediating EMT-like changes during tissue regeneration.

## 2. Results

### 2.1. Antibody Microarray Analysis of the Differentially Expressed Proteins in Telomerase-Immortalized Human Corneal Epithelial (hTCEpi) Cells

**Figure 1:**
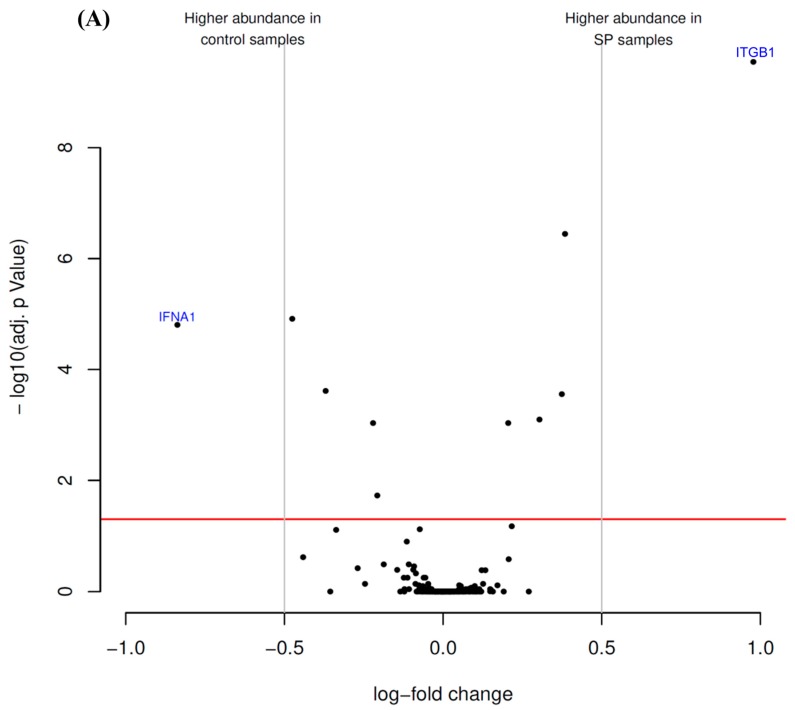

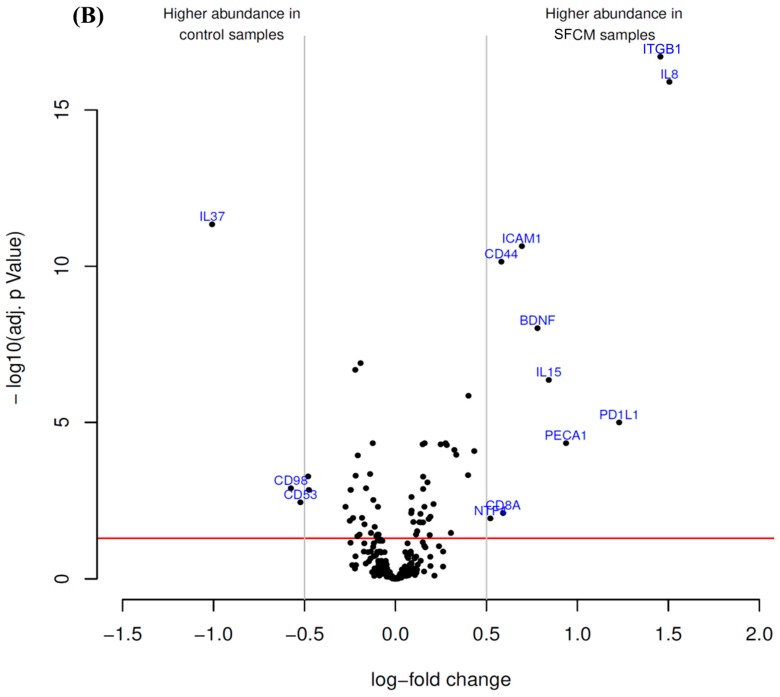
Antibody microarray identified differentially enriched proteins after the treatment of hTCEpi cells with (**A**) substance P (SP) and (**B**) stromal fibroblasts conditioned medium (SFCM). The volcano plot visualizes the *p*-values (adjusted for multiple testing) and corresponding log-fold changes. The log-fold change of the difference in abundance is shown horizontally whereas the vertical axis indicates the significance level. The black dots represent the analyzed proteins. The horizontal red line indicates an adjusted *p*-value of 0.05, above which all proteins are considered to vary significantly. Proteins with a positive log-fold change had a higher abundance in SP or SFCM samples whereas proteins with a negative value in control samples.

Antibody microarrays were employed to identify putative alterations in the expression pattern of CD markers and cytokines by hTCEpi cells after treatment with either SP or SFCM (Figure 1).

The presence of SP in incubation medium suppressed the level of interferon-α1 (IFNA1) and potently stimulated the expression of integrin β1 (ITGB1) in comparison with control cells (Figure 1A; Table 1).

Antibody microarray analysis of hTCEpi cell lysates revealed that the treatment of cells with SFCM affected the expression of 13 proteinous products (Figure 1B). Among them, interleukin-8 (IL-8), ITGB1, programmed cell death 1 ligand 1 (PD1L1 or CD274), platelet endothelial cell adhesion molecule (PECA1 or CD31), interleukin-15 (IL-15), brain-derived neurotrophic factor (BDNF), intercellular adhesion molecule 1 (ICAM1 or CD54), T-cell surface glycoprotein CD8 α chain (CD8A), CD44, neurotrophin-4 (NTF4) were abundantly present in SFCM treated samples. In contrast, leukocyte surface antigen CD53 (CD53), 4F2 cell-surface antigen heavy chain (CD98), and interleukin-37 (IL-37) expression was suppressed in comparison with the control cells (Figure 1B, Table 2).

The characteristics of the identified proteinous compounds with altered expression stimulated by SFCM, with respect to their subcellular localization (Table 3) and molecular function (Table 4) were obtained from STRING database. Similarly, also the lists of the biological pathways (Table 5) and processes (Table 6) were generated. The hierarchial cluster analysis data (Figure S1) and the distribution of individual array values of some proteins (Figure S2) were shown in the Supplementary Information.

### 2.2. Treatment of Corneal Epithelial Cells with SP and SFCM Activates Vital Signaling Molecules

**Figure 2:**
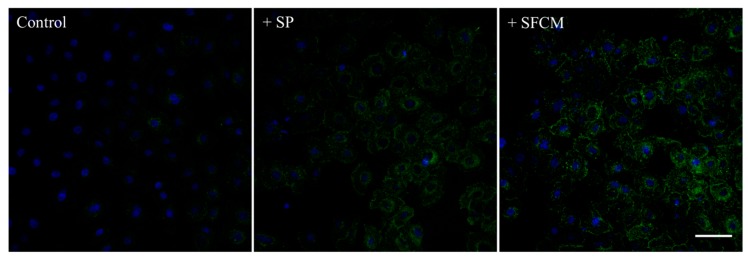
Immunofluorescence detection of the total protein tyrosine phosphorylation in hTCEpi cells after treatment with SP and SFCM. Growth factor-starved hTCEpi cells were cultured in the presence of SP and SFCM for 24 h and the protein tyrosine phosphorylation was analyzed by staining with an anti-phosphotyrosine (green) antibody. An increase in the total protein tyrosine phosphorylation was observed in hTCEpi cells treated with SP and SFCM. Nuclei were counterstained with 4,6-diamidino-2-phenylindole (DAPI) (blue). Scale bar: 50 μm.

Based on our antibody microarray results, we further intended to study activated signaling pathways upon the addition of SP and SFCM in hTCEpi cells. An increased total protein tyrosine phosphorylation in hTCEpi cells treated with SFCM was observed than with SP (Figure 2).

**Figure 3:**
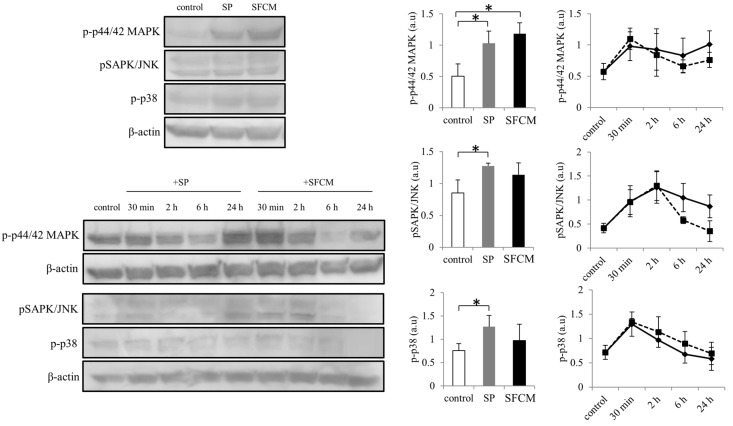
Activation of mitogen-activated protein kinases (MAPK) signaling pathway following SP and SFCM treatment in hTCEpi cells. After stimulation, protein lysates were collected at time points 30 min, 2, 6, and 24 h and subjected to immunoblot analysis. Similarly, protein lysates collected directly after 24 h were also used. Phosphorylation of p44/42 MAPK, SAPK/JNK and p38 proteins were analyzed using respective antibodies. Corresponding β-actin protein levels were used to compare and calculate the differences in the phosphorylation levels. Data represent the mean of the phosphorylation levels (*n* > 3) shown as arbitrary units. Bar graphs indicate the mean phosphorylation levels after 24 h of treatment with SP and SFCM. In the line graphs, straight lines (―) indicate SP stimulation time points and dotted lines (…) indicate time points after SFCM stimulation. The *p*-values of <0.05 were considered statistically significant and are indicated by asterisks (*).

**Figure 4:**
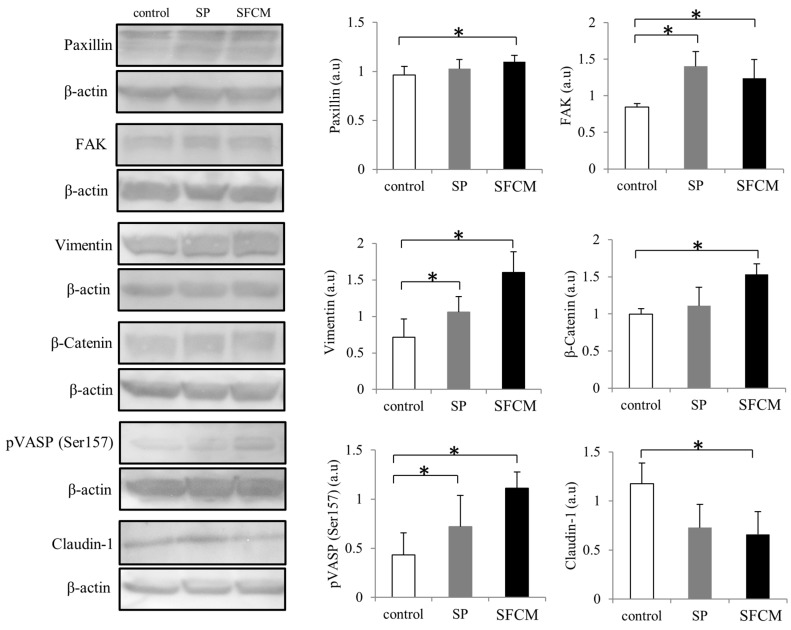
Activation of important signaling molecules paxillin, focal adhesion kinase (FAK), vimentin, β-Catenin, phosphorylation of vasodilator-stimulated phosphoprotein (VASP) and claudin-1 following SP and SFCM stimulation in hTCEpi cells. Protein lysates were collected after 24 h of stimulation and subjected to immunoblot analysis. Relative expression levels of the individual proteins were analyzed using respective antibodies. Corresponding β-actin protein levels were used to compare and calculate the differences in the expression levels. Data represent the mean of the expression levels (*n* > 3) shown as arbitrary units. Bar graphs indicate the mean expression levels after 24 h of treatment with SP and SFCM. The *p*-values of <0.05 were considered statistically significant and are indicated by asterisks (*).

Activation of MAPK family proteins within 30 min after the addition of SP and SFCM was observed. After 24 h of stimulation, increased phosphorylation of p44/42 MAPK, SAPK/JNK, and p38 kinases were observed in the presence of SP whereas phosphorylation of only p44/42 MAPK and p38 was observed in the presence of SFCM (Figure 3). Furthermore, we also analyzed the effect of SP and SFCM on the expression of important key signaling molecules related to focal adhesions, actin reorganization and EMT (Figure 4). A significant increase in the expression of paxillin was observed in the presence of SFCM. Under the both treatment conditions, either in presence of SP or SFCM, the enhanced focal adhesion kinase (FAK) and vimentin expression were observed (Figure 4).

**Figure 5:**
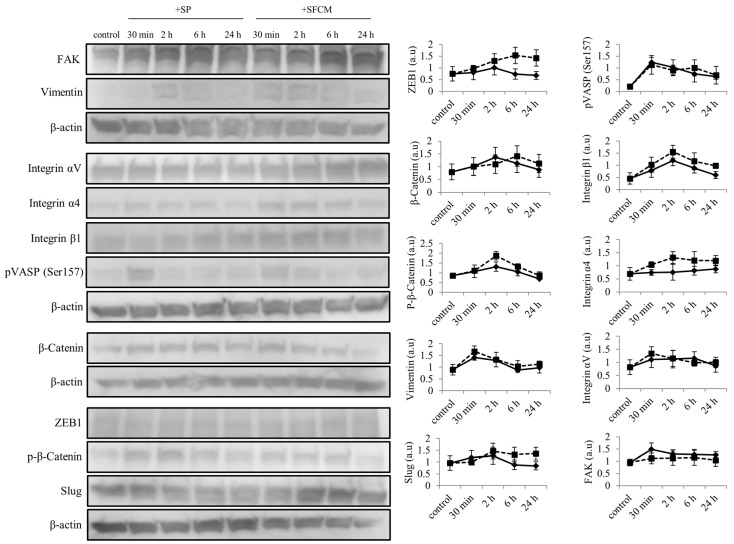
Differences in the expression of various important signaling molecules (ZEB1, phosphorylation of VASP, β-Catenin, integrin β1, phosphorylation of β-Catenin, integrin α4, vimentin, integrin αV, Slug and FAK) at different time points following SP and SFCM stimulation in hTCEpi cells. After stimulation, protein lysates were collected at time points 30 min, 2, 6, and 24 h and subjected to immunoblot analysis. Relative expression levels of the individual proteins were analyzed using respective antibodies. Corresponding β-actin protein levels were used to compare and calculate the differences in the expression levels. Data represent the mean of the expression levels (*n* > 3) shown as arbitrary units. In the line graphs, straight lines (―) indicate SP stimulation time points and dotted lines (…) indicate time points after SFCM stimulation.

After the addition of SP, increased expression of FAK and vimentin were observed, whereas in case of SFCM treatment the expression of FAK was gradually increased comparing to vimentin. Levels of β-catenin were considerably enhanced in both, SP and SFCM treated cells, until 24 h whereas phosphorylated β-catenin levels were gradually decreased (Figure 5). Another important signaling molecule significantly activated in epithelial cells after the addition of SP and SFCM is vasodilator-stimulated phosphoprotein (VASP). We observed an enhanced and constitutive phosphorylation of VASP at Ser157 until 24 h (Figure 4 and Figure 5). Moreover, the presence of SP and SFCM significantly reduced the expression of tight junctional protein claudin-1 in epithelial cells (Figure 4). In addition, the expression of EMT-promoting transcription factors zinc finger E-box binding homeobox 1(ZEB1) and Slug also varied in SFCM treated cells than in SP treated cells (Figure 5). Slug expression reached its peak after 2 h of SP stimulation and reached to normal levels within 6 h. In contrast, SFCM addition leads to the increased expression levels of Slug after 2 h which was sustained until 24 h. Similar to Slug expression, SP stimulated ZEB1 expression also enhanced slightly after 2 h and reached to normal levels within 6 h whereas SFCM stimulated ZEB1 expression increased gradually until 6 h and sustained until 24 h.

### 2.3. Activation of Integrin Signaling

**Figure 6:**
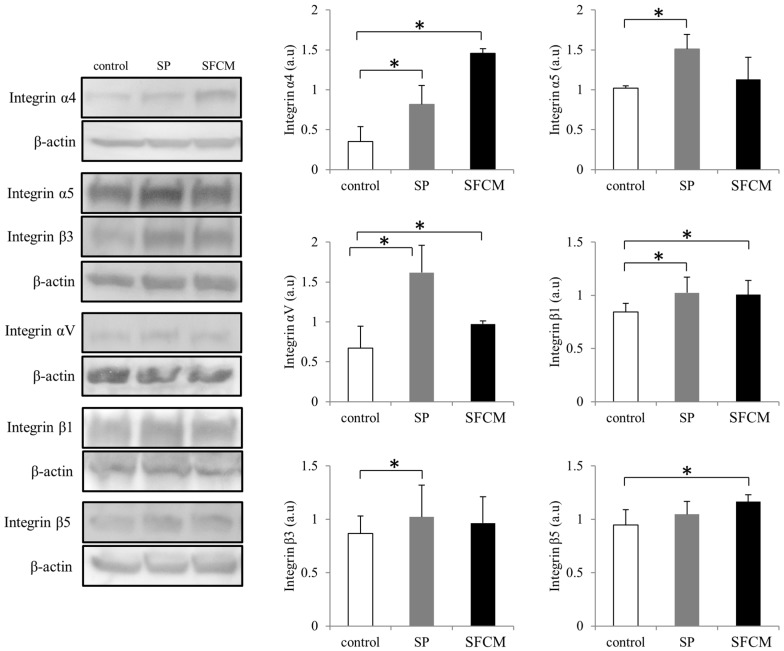
Differences in the expression of various integrin subunits (α4, α5, αV, β1, β3 and β5) following SP and SFCM stimulation in hTCEpi cells. Protein lysates were collected after 24 h of stimulation and subjected to immunoblot analysis. Relative expression levels of the individual proteins were analyzed using respective antibodies. Corresponding β-actin protein levels were used to compare and calculate the differences in the expression levels. Data represent the mean of the expression levels (*n* > 3) shown as arbitrary units. Bar graphs indicate the mean expression levels after 24 h of treatment with SP and SFCM. The *p*-values of <0.05 were considered statistically significant and are indicated by asterisks (*).

ITGB1 is the only molecule that was found to be abundantly and commonly expressed in corneal epithelial cells after the treatment with either SP or SFCM during antibody microarrays. To further understand the role of other integrins in corneal wound healing, we studied differences in the expression of various integrins (Figure 5 and Figure 6). In the presence of SP, we observed a significant increase in the expression of α4, α5, αV, β1 and β3 subunits (Figure 6). Similarly, SFCM also enhanced the expression of integrin subunits α4, α5, αV, β1 and β5 (Figure 6). Integrin β1 expression was reached its maximum after 2 h of the addition of SP and SFCM to the epithelial cells (Figure 5). Even though its expression decreased gradually, after 24 h its levels were still higher than the control. Integrin α4 expression was gradually and slightly increased during SP treatment, whereas SFCM stimulated increase in α4 integrin reached its maximum levels in 2 h and was persistent until 24 h (Figure 5 and Figure 6).

**Figure 7:**
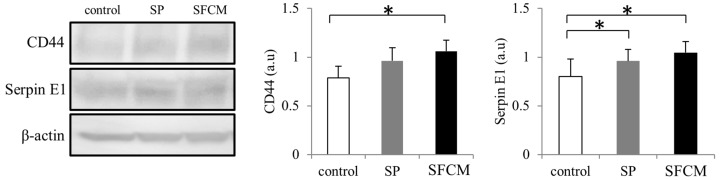
Differences in the expression of CD44 and Serpin E1 following SP and SFCM stimulation in hTCEpi cells. Protein lysates were collected after 24 h of stimulation and subjected to immunoblot analysis. Relative expression levels of the individual proteins were analyzed using respective antibodies. Corresponding β-actin protein levels were used to compare and calculate the differences in the expression levels. Data represent the mean of the expression levels (*n* > 3) shown as arbitrary units. Bar graphs indicate the mean expression levels after 24 h of treatment with SP and SFCM. The *p*-values of <0.05 were considered statistically significant and are indicated by asterisks (*).

We also observed considerably enhanced CD44 expression in our cultured epithelial cells following the addition of SFCM (Figure 7). Additionally, increased expression of Serpin E1, another important injury-response molecule, was also observed in the presence of SP and SFCM (Figure 7). 

### 2.4. SFCM Enhances Epithelial Cell Motility and Migration

**Figure 8:**
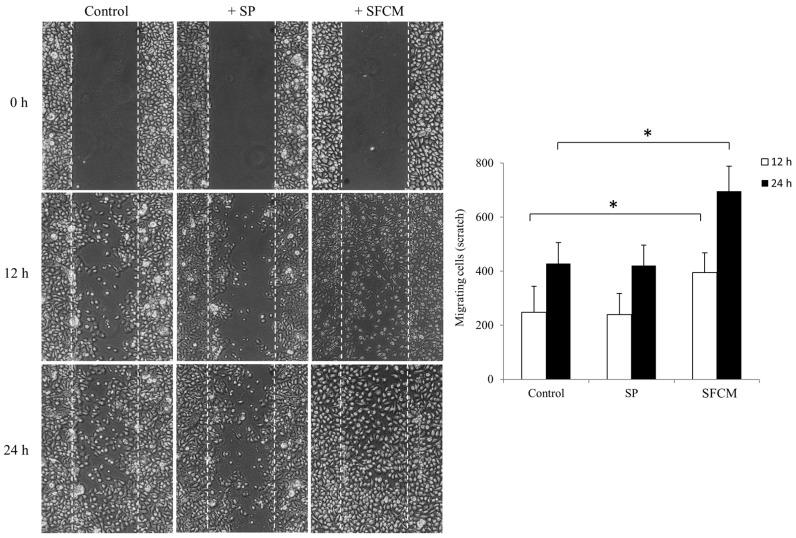
Wound healing scratch assay was made on 24 h growth factor-starved, confluent hTCEpi cells by scratching a line across the bottom of the culture dish. SP and SFCM were added to the culture media and the cell motility and migration was observed at time points 12 h and 24 h, respectively. The micrographs show the extent of scratch closure obtained under control conditions compared to those with the addition of SP and SFCM. Cell migration quantification was evaluated by counting the number of cells in the central gap. Three independent experiments were performed and a representative result is shown. The *p*-values of <0.05 were considered statistically significant and are indicated by asterisks (*).

To further study the role of SP and SFCM in corneal epithelial cell motility and migration, we performed a scratch assay. The presence of SFCM enhances motility and migration of corneal epithelial cells, in comparison to the control and SP treated cells (Figure 8). During wound healing assay, SFCM treated corneal epithelial cells filled the scratch area much faster than untreated control and SP treated cells. Cell migration was represented as a number of cells filling the central gap area after making the scratch. We observed a significant increase in the number of migrating cells in the presence of SFCM in both conditions, 12 h as well as 24 h, compared to the control and SP treated cells after making the scratch (Figure 8). We believe that addition of SFCM increases also cell proliferation [8] along with motility and migration in comparison to the SP or untreated control cells.

## 3. Discussion

Epithelial-mesenchymal cell communications are essential to regulate wounded tissue regeneration following injury [33,34]. Similarly, in the cornea, cell–cell interactions play dominant role throughout the process of wound healing [35], in which cytokine, growth factor and chemokine-mediated stromal-epithelial interactions are indispensable [2,3,5,36,37,38,39,40]. Additionally, conditioned media from the corneal stromal cells is also known to stimulate epithelial cell growth, proliferation and nerve regeneration due to the presence of various cytokines, keratins, growth factors, chemokines, neurotrophic factors and vital signaling molecules [7,8,9,41]. The present work was focused on to unravel the functional molecules that are necessary to promote cell growth, motility and proliferation of wounded corneal epithelial cells. Furthermore, we explored this aspect, in the context of epithelial-stromal interactions, using antibody microarrays to identify differentially regulated cell surface markers and cytokines that are essential for the epithelial cell function. Since SFCM contains surplus essential factors, including neuropeptide protachykinin-1, than stromal keratocyte conditioned medium [9], we employed CM collected from the SFs alongside SP in the present study to investigate their trophic effects on corneal epithelial cells.

Antibody microarray analysis revealed a higher abundance of ITGB1 in SP treated cells, which plays an important role in cell-ECM adhesion, migration, tissue repair [42,43] and its appearance in the cornea was correlated with the process of corneal epithelial wound repair [29]. Treatment of epithelial cells with SFCM resulted to the differential expression of several proteins that have either direct or indirect roles in epithelial cell growth, movement, and adhesion-related signaling cascades during tissue regeneration. Similar to the SP treatment, we also observed upregulated ITGB1 in SFCM treated corneal epithelial cells. IL-8 contributes to cell migration and chemotaxis during tissue repair [44,45,46]. IL-8 release from the cells is mainly regulated at the transcriptional level and MAPK pathway plays an important role in stabilizing IL-8 mRNA and its protein expression [47,48]. Since trophic factors in the CM also regulate IL-8 synthesis [49], the observed increase in IL-8 production after the addition of SFCM may be due to the increased stability of IL-8 mRNA facilitated in part by the trophic factors in SFCM together with SFCM-mediated MAPK pathway activation. CD274 modulates the corneal immune response and functions as an anti-angiogenic molecule [50,51] and its up-regulation after the addition of SFCM may contribute to protect immune privilege status of the wounded cornea during tissue renewal. Cell surface molecule CD31 participates in governing cell adhesion and activation of key signal transduction cascades by recruiting adaptor proteins [52,53]. Pro-inflammatory cytokine IL-15 promotes cell proliferation, migration, and regeneration in response to injury [54,55,56] and also stimulates the production of IL-8 [57]. The collective production of IL-15 and IL-8 following the addition of SFCM may induce EMT-like changes [58,59] to augment epithelial cell migration and tissue regeneration following injury. CD54 is upregulated in the healing corneas and participate in the epithelial recruitment of T cells along with enhanced cell motility and migration [60,61,62]. Cells lacking T-cell receptor also express CD8A and its expression is known to be modulated by cytokines [63,64,65]. CD44 is a predominant cell surface glycoprotein receptor and adhesion molecule which plays an important role in signal transduction processes related to cell adhesion, wound healing, EMT [66,67,68,69] and its expression correlates with re-epithelialization in the healing cornea [70]. The nerve growth factor family of neurotrophins including BDNF and NTF4 mediate trophic properties and are highly expressed during corneal nerve regeneration [71,72,73]. They promote neurite outgrowth and necessary for the survival of axotomized neurons [74,75,76] as well as growth and proliferation of non-neuronal cells by activation of MAPK signaling pathway [77,78,79]. SFCM induced expression of BDNF, NT4 may also promote epithelial cell proliferation in conjunction with corneal nerve regeneration after injury and further emphasize the interdependence of stromal-epithelial cell alliance in corneal nerve regeneration. Protein–protein interaction analysis, following the treatment of corneal epithelial cells with SFCM, revealed distinct direct as well as indirectly interconnected networks among the abundantly expressed cell surface molecules and cytokines (Figure S3), additionally highlights the prominence of stromal–epithelial interactions during corneal epithelial tissue repair.

Coordinated collective cell migration through sustained intercellular connections is the fundamental cell movement during corneal epithelial wound repair in vivo [80,81,82] where adhesive cell-substratum interactions, including cell–cell and cell–ECM adhesions, are mandatory for sustained cell motility and migration towards the injured site [36,83,84]. During in vitro scratch-wound healing models, lamellipodial crawling is the most commonly observed migration mechanism where each polarized cell progress through different structural alterations that include lamellipodium formation, nucleus translocation and trailing edge detachment [82,85]. This mechanism is further regulated by complex events of actin polymerization, depolymerization, and integrin signaling [86,87]. Correspondingly, alteration of plasticity plays an indispensable part in the process of EMT during which cells lose their epithelial characteristics without impairing the cell-cell interactions associated with collective migration and acquire mesenchymal qualities associated with individual migration in a reversible manner [88,89,90]. This transition further involves synchronized alterations in various additional cell structures including actin cytoskeleton reorganization, engagement, and expression of various integrins which navigate cells through the ECM [88,91,92]. During the course of tissue repair, all these coordinated processes are in part executed by complex and overlapping signaling networks involving growth factors, cytokines, and chemokines [93].

Reorganization of the cytoskeleton along with actin filament remodeling at the leading edge is essential for EMT and cell migration during epithelial injury. VASP mediated focal adhesion, actin filament binding and polymerization inhibits cell movement whereas its phosphorylation inhibits actin polymerization [94] and thereby induces epithelial cell motility during repair along with alteration of various protein–protein interactions [95,96]. The observed consistent phosphorylation of VASP at Ser157, but not at Ser239, in corneal epithelial cells in the presence of SFCM and SP may assist in locating VASP at the leading edge to induce filopodia formation [97] during cell motility. Additionally, phosphorylated VASP mediated actin remodeling events dismantle cell–cell junctions during the progression of EMT [98,99]. During cell migration, protein tyrosine kinase FAK acts downstream of different growth factor and ECM components and is a central player of integrin-mediated motility [100]. Furthermore, it activates focal adhesion signaling proteins and regulates cell adhesion disassembly through adopter protein paxillin [101]. In our study, after stimulation of cells with SP and SFCM, we also observed increased expression of FAK, paxillin along with steady activation of p44/42 MAPK and integrin β1. Since ERK (Extracellular signal-regulated kinase)-FAK-paxillin pathway regulates cell migration along with the recruitment of integrin β1 [102,103], we also presume the activation of ERK-FAK-paxillin-integrin β1 cascade in the presence of SP and SFCM during corneal epithelial wound repair. Vimentin functions as a signal integrator during EMT, wound repair and also regulates structure and function of focal adhesions by linking its intermediate filaments to paxillin [104,105,106,107]. Epithelial vimentin expression during the corneal wound repair is linked to cell–matrix interactions and cell migration [108]. In addition, vimentin also binds to p44/42 MAPK and prolongs its activation by preventing dephosphorylation [109]. The vimentin-p44/42 MAPK axis further regulates the transcriptional activity of Slug to establish and promote EMT-like alterations [110]. As the expression of vimentin, p44/42 MAPK correlates with transcription factor Slug expression in the course of EMT, their consistent activation in the presence of SFCM may also indicate the incidence of EMT-like modifications during epithelial tissue repair.

Integrins are heterodimeric proteins composed of transmembrane α, β subunits and are important for epithelial cell migration during corneal tissue regeneration [31]. Integrin expression is regulated by cytokines and growth factors [111]. Integrin β1expression is enhanced during corneal wound repair in actively migrating epithelial cells [29], and its deletion leads to unstable adhesions and delayed wound healing [112]. Integrin subunits αV, β1 and β5 mediate interactions with ECM proteins and migration [31,113] whereas integrin α4 promotes motility and serves as EMT marker protein [114,115] and α5 triggers β-catenin mediated EMT and migration [116]. Integrin αV and β3 contributes to the TGF-β mediated EMT process through FAK and ERK-mediated pathways by dissociating cell–cell junctions [27,28,117]. Furthermore, SP-induced effects were mediated by αV, β3 subunits [118] and SP enhances α5 expression [119]. Similarly, in this study, we also observed increased expression of α5, αV, as well as β3 integrins confirming SP facilitated influence on epithelial cells. SFCM facilitated upregulation of migration-associated α4, α5, αV, β1 and β5 integrins may play a role in inducing the migration of epithelial cells as we observed in our scratch assay. Furthermore, by modulating the function of integrins and ECM, SerpinE1 also induce cell migration along with EMT during wound healing [120,121]. Since vimentin binding to integrin cytoplasmic tails regulate integrin-ligand interactions [122] which in turn propagates through MAPK pathway [123,124], the observed enhanced expression of EMT markers, migration-associated integrins in conjunction with activated ERK-FAK-paxillin axis may have an influence on SFCM mediated cell proliferation and migration in this study.

The zinc finger transcription factors Slug and ZEB1 regulate cell migration and basic events of EMT [20]. Slug regulates transcriptional activation of ZEB1, controls the expression of adhesion molecules and integrins together with suppression of epithelial claudin-1 expression [125,126,127]. Similarly, ZEB1 also involved in the activation of focal adhesion formation and cytoskeletal remodeling events [128,129]. In corneal epithelial cells we also observed Slug dependent expression of ZEB1. Increased Slug levels were correlated with increased ZEB1 levels during SFCM stimulation which was lasted until 24 h. In addition, the decreased claudin-1 levels also correlate with Slug in SFCM treated epithelial cells which may sequentially lead to the onset of EMT-like transition [130]. The key player of canonical Wnt signaling, β-catenin, is also expressed in the corneal epithelium where its activation escalates epithelial cell proliferation and EMT [20,131]. Similarly, elevated β-catenin protein levels were detected during wound healing [132,133]. Non-phosphorylated β-catenin translocates to the nucleus and activates various transcription factors whereas Ser33, Ser37, and Thr41 phosphorylated form targeted for ubiquitin-mediated proteasomal degradation. As Slug is also known to promote the activation of β-catenin [134], sustained β-catenin in conjunction with activated integrins and Slug [135] may also shift the equilibrium towards cell migration and contribute to type 2 EMT-like alterations in the epithelial cells following the addition of SFCM.

SP treatment alone is insufficient to promote injury induced corneal epithelial cell migration [136,137] and SP-mediated effects are mediated in synergy with other growth factors [136,137,138,139] during corneal wound healing. In accordance with these observations, we also did not observe any SP-mediated cell migration during our scratch assay. Instead, the SP-mediated activation of MAPK signaling pathway, integrins, and other key signaling molecules that we observed in our study may contribute to the corneal epithelial sensitization, homeostasis and wound repair, in synergy with other growth factors [139,140,141]. Furthermore, the enhanced proliferation and migration of epithelial cells upon the addition of SFCM correlates with the presence of numerous amounts of trophic factors in the stromal conditioned medium [9]. Taken together, SFCM mediated enhanced and sustained activation of ZEB1, Slug transcription factors in combination with upregulated migration-associated integrins and ERK-FAK-paxillin axis, may lead to induce type 2 EMT-like changes during wound healing. Based on our results, we conclude that stromal-epithelial interactions, by activating various interconnected cell surface molecules, growth factors and cytokines, may control key intracellular signaling events that are crucial to promote EMT-like changes along with nerve regeneration during tissue repair at the ocular surface.

## 4. Materials and Methods

### 4.1. Materials

The following antibodies used in the present study were purchased from Cell Signaling Technology (Frankfurt, Germany); Phospho-tyrosine (Cat no. 9411), phospho-SAPK/JNK (Cat no. 4668), phospho-p38 MAPK (Cat no. 4511), phospho-p44/42 MAPK (Cat no. 4370), claudin-1 (Cat no. 13255), TCF8/ZEB1 (Cat no. 3396), phospho-VASP (Ser157) (Cat no. 3111), vimentin (Cat no. 5741), β-Catenin (Cat no. 8480), phospho-β-Catenin (Ser33/37/Thr41) (Cat no. 9561), Slug (Cat no. 9585), FAK (Cat no. 3285), paxillin (Cat no. 12065), integrin α4 (Cat no. 8440), integrin α5 (Cat no. 4705), integrin αV (Cat no. 4711), integrin β1 (Cat no. 9699), integrin β3 (Cat no. 13166), integrin β5 (Cat no. 3629). CD44 (Cat no. MA5-13890) was from Thermo Fischer (Darmstadt, Germany), Serpin E1 (Cat no. MAB1786) was purchased from R&D systems (Wiesbaden-Nordenstadt, Germany) and β-actin (Cat no. A5316) antibody was from Sigma-Aldrich (Munich, Germany). Anti-rabbit IgG- HRP antibody (Cat no. 7074) and anti-mouse IgG-HRP antibody (Cat no. 7076) were purchased from Cell Signaling Technology (Frankfurt, Germany). TPP tissue culture flasks, dishes and 6, 12-well plates were obtained from Sigma-Aldrich. Coverslips and glass slides were from Marienfeld (Bonn, Germany). Enhanced chemiluminescence (ECL) prime detection western blotting reagent was purchased from Amersham (Munich, Germany) and other western blotting reagents were from Bio-Rad (Munich, Germany). Substance P (SP) (Cat no. 1156) was purchased from Tocris (Wiesbaden-Nordenstadt, Germany).

### 4.2. Cell Culture

This study was approved by the ethics committee of the University of Rostock (Approved ID: A 2014-0100) and followed the guidelines of the Declaration of Helsinki.

In this present study, hTCEpi cell-line [142], between passages 20–28, was used (a kind gift of James V Jester; authenticated and characterized according to ATCC standard protocols). hTCEpi cells were cultured in KGM-Gold™ growth medium (Lonza, Köln, Germany). Cells were sub-cultured on T75 tissue culture flasks (Sigma-Aldrich), incubated at 37 °C in 5% CO_2_ and passaged every 5 to 7 days.

Primary human SFs were cultured using an explant culture method, after collecting corneas from donor cadavers, in DMEM with low glucose (Sigma-Aldrich) supplemented with 10% fetal calf serum. SFs were seen growing out of the corneal explants after 3–4 days. When outgrowing primary SFs reached a confluent monolayer, cells were trypsinized and subcultured. For experiments in this present study, SFs of third passage were used. The mesenchymal origin of the cells was confirmed by immune staining with antibody against vimentin.

For the collection of CM from the cultured primary SFs, KGM-Gold medium without any supplemented growth factors was added to the phosphate buffered salinePBS washed confluent cultures and the respective SFCM was collected after 24 h to stimulate hTCEpi cells immediately (3:1 dilution with normal KGM-Gold medium without supplemented growth factors) or stored at −70 °C. The collected SFCM was spun at 300× *g* for 5 min to remove any remaining cell debris. During stimulation of hTCEpi cells, confluent cultures were growth factor-starved for 24 h before stimulation and SP was added at the concentration of 10^−5^ M along with the growth factor-deprived cell culture media.

### 4.3. Antibody Microarray Analysis

To analyze the differential expression of CD markers and cytokines (scio CD—Cell surface marker and Cytokine profiling) in hTCEpi cells in the presence of SFCM and SP, cells were treated with SFCM and SP, for 24 h as described above. Later, the cells were collected, washed and frozen cell pellets were sent to Sciomics GmbH (Heidelberg, Germany) for further analysis. For each condition, the array was performed in triplicates. Briefly, proteins were extracted, quantified and labeled with fluorescent dyes. All nine samples were analyzed in a dual-color approach using a reference-based design on scioCD antibody microarrays (Sciomics) targeting 95 different CD surface markers and 26 cytokines/chemokines with 270 different monoclonal antibodies. The list of the analyzed target proteins was provided as supplementary information. Each antibody is represented in eight replicates on the array. The arrays were blocked with scioBlock (Sciomics) on a Hybstation 4800 (Tecan, Grödig, Austria). Resulting data were analyzed using the linear models for microarray data (LIMMA) package of R-Bioconductor after uploading the median signal intensities for differential protein expression. For normalization, a specialized invariant Lowess method was applied and for analysis of the samples, a one-factorial linear model was fitted with LIMMA resulting in a two-sided *t*-test or F-test based on moderated statistics. All presented *p* values were adjusted for multiple testing by controlling the false discovery rate according to Benjamini and Hochberg. Proteins were defined as differential for |logFC| > 0.5 and an adjusted *p*-value < 0.05. Differences in protein abundance between different samples or sample groups are presented as log-fold changes (logFC) calculated for the basis 2. In a study comparing samples versus control a logFC = 1 means that the sample group had on average a 2^1^ = 2-fold higher signal as the control group. logFC = −1 stands for 2^−1^ = 1/2 of the signal in the sample as compared to the control group.

### 4.4. Immunofluorescence

Epithelial cells grown on glass coverslips were fixed with paraformaldehyde (4%, *w*/*v* in PBS) for 10 min after washing with PBS and then permeabilized with PBS containing 0.1% Triton X-100 (Sigma-Aldrich) for 30 min. Fixed and permeabilized cells were then incubated with primary antibody for intracellular staining. Primary antibody in this study was used at 1:100 dilution in PBS containing 0.1% Triton X-100 with 2% FCS and incubated for 60 min at room temperature. The cells were then washed with PBS before adding secondary donkey anti-mouse IgG (H + L)-Alexa Fluor 488 (diluted 1:500) and incubated at room temperature for another 60 min. Later, cells were washed 3 times with PBS and mounted in mounting medium (Vector Labs, Eching, Germany) containing 4,6-diamidino-2-phenylindole (DAPI). Cells were observed under a Nikon confocal fluorescence microscope equipped with a digital camera (Nikon Eclipse E400 with D-Eclipse C1) (Dusseldorf, Germany) and all images were taken from a single plane through the cell monolayers with 40× objective using the same settings.

### 4.5. Immunoblotting

After treatment with SFCM or SP, hTCEpi cell monolayers were washed with PBS and lysed in RIPA buffer (Sigma-Aldrich) containing protease and phosphatase inhibitors (Roche, Mannheim, Germany). Equal amounts of total cell lysates were loaded into the wells of 10% Mini-PROTEAN^®^ TGX™ precast gels (Bio-Rad), separated by SDS-PAGE and transferred onto PVDF membranes (Bio-Rad). Later, membranes were blocked with 5% non-fat dry milk (Carl Roth, Karlsruhe, Germany) in Tris-buffered saline with 0.1% Tween-20 (TBS-T) for 30 min and incubated with respective primary antibodies (diluted 1:1000) at 4 °C overnight. After washing 3 times with TBS-T, membranes were incubated with secondary HRP-conjugated anti-rabbit or anti-mouse IgG (diluted 1:2500) for an additional 1 h at room temperature and developed to visualize protein bands using the *ECL* detection system. During quantification, the optical density of each protein band was normalized to the corresponding β-actin band. Quantification of the blots was performed using ImageJ software [143].

### 4.6. Scratch Wound Healing Assay

Migration of wounded corneal epithelial cells was assessed by performing an in vitro scratch assay in which a linear wound midline was made across the bottom of the dish on a confluent monolayer of epithelial cells using a 200 µL sterile pipet tip. After that, cells were rinsed gently with PBS to remove any remaining cell debris. KGM-Gold™ medium deprived of growth factors was used during the scratch assay. SFCM and SP were used as mentioned in Section 2.2. Micrographs of the cells were taken at 10× magnification using a microscope equipped with a Moticam10 digital camera at time points 0, 12 and 24 h. Epithelial cell migration across the wound line was then quantified by counting the cells invading the central scratch area in the control, SP and SFCM treated culture dishes.

### 4.7. Statistical Analyses

Bar charts and line plots were generated using means and the standard deviation. Student’s *t*-test was used for the comparison between two groups (control vs. SP; control vs. SFCM) and *p*-values of <0.05 were considered statistically significant and are indicated by asterisks.

## Figures and Tables

**Table 1:** Proteins with most profound differential expression in hTCEpi cells after treatment with substance P (SP).

Protein (Human)	logFC	Average Expression	Adjusted *p*-Value	UniProt Access ID
ITGB1	0.98	13.87	2.8 × 10^−10^	P05556
IFNA1	−0.84	10.23	1.6 × 10^−5^	P01562

Proteins with a positive logFC value had a higher abundance in SP treated samples, whereas proteins with a negative value in control samples. Average expressions as well as *p*-values adjusted for multiple testing are listed.

**Table 2:** Proteins with most profound differential expression in hTCEpi cells after treatment with stromal fibroblasts conditioned media (SFCM).

Protein (Human)	logFC	Average Expression	Adjusted *p*-Value	UniProt Access ID
IL-8	1.51	13.39	1.3 × 10^−16^	P10145
ITGB1	1.46	13.87	2.0 × 10^−17^	P05556
PD1L1	1.23	11.93	1.0 × 10^−5^	Q9NZQ7
PECA1	0.94	11.72	4.6 × 10^−5^	P16284
IL-15	0.84	8.29	4.4 × 10^−7^	P40933
BDNF	0.78	8.73	9.6 × 10^−9^	P23560
ICAM1	0.69	14.76	2.3 × 10^−11^	P05362
CD8A	0.59	10.97	8.0 × 10^−3^	P01732
CD44	0.58	12.18	7.2 × 10^−11^	P16070
NTF4	0.52	8.81	1.2 × 10^−2^	P34130
CD53	−0.52	10.34	3.6 × 10^−3^	P19397
CD98	−0.57	11.96	1.3 × 10^−3^	P08195
IL-37	−1.01	13.40	4.6 × 10^−12^	Q9NZH6

Proteins with a positive logFC value had a higher abundance in SFCM treated samples, whereas proteins with a negative value in control samples. Average expressions, as well as *p*-values adjusted for multiple testing, are listed.

**Table 3:** Location of antibody microarray identified, highly enriched proteins in hTCEpi cells after treatment with SFCM.

GO Pathway ID	Cellular Component	Protein Count	Matching Proteins
GO.0009897	external side of plasma membrane	5	CD274, CD44, CD8A, ICAM1, ITGB1
GO.0005576	extracellular region	8	CD274, CD44, CD8A, ICAM1, IL15, IL8, ITGB1, NTF4
GO.0044421	extracellular region part	7	BDNF, CD274, CD44, ICAM1, IL15, IL8, ITGB1
GO.0005887	integral component of plasma membrane	5	CD44, CD8A, ICAM1, IL15, ITGB1
GO.0043235	receptor complex	3	CD44, CD8A, ITGB1

Analysis was performed using the STRING database, version 10.5 (Available online: http://string-db.org). (GO = gene ontology).

**Table 4:** Annotated molecular function of the antibody microarray identified highly abundant proteins in hTCEpi cells after treatment with SFCM.

GO Pathway ID	Molecular Function	Protein Count	Matching Proteins
GO.0005102	receptor binding	6	BDNF, CD8A, ICAM1, IL15, IL8, NTF4

Analysis was performed using the STRING database, version 10.5 (Available online: http://string-db.org). (GO = gene ontology).

**Table 5:** Significantly impacted signaling pathways in hTCEpi cells after SFCM stimulation.

KEGG Pathway ID	Pathway Description	Protein Count	Matching Proteins
4514	Cell adhesion molecules (CAMs)	4	CD274, CD8A, ICAM1, ITGB1
4064	NF-kappa B signaling pathway	2	ICAM1, IL8
4512	ECM-receptor interaction	2	CD44, ITGB1
4640	Hematopoietic cell lineage	2	CD44, CD8A
4668	TNF signaling pathway	2	ICAM1, IL15
4670	Leukocyte transendothelial migration	2	ICAM1, ITGB1
4722	Neurotrophin signaling pathway	2	BDNF, NTF4

After antibody microarray, highly enriched proteins function and their interactions with other proteins was analyzed to identify impacted signaling pathways using the STRING database, *version 10.5* (Available online: http://string-db.org). (KEGG = Kyoto Encyclopedia of Genes and Genomes).

**Table 6:** Significantly impacted biological processes in hTCEpi cells during SFCM stimulation.

GO Pathway ID	Biological Process	Protein Count	Matching Proteins
GO.0006954	inflammatory response	5	BDNF, CD44, ICAM1, IL15, IL8
GO.0030155	regulation of cell adhesion	5	CD274, CD44, ICAM1, IL15, IL8
GO.0050900	leukocyte migration	4	CD44, ICAM1, IL8, ITGB1
GO.0007159	leukocyte cell-cell adhesion	4	CD44, ICAM1, IL15, ITGB1
GO.0002682	regulation of immune system process	6	CD274, CD8A, ICAM1, IL15, IL8, ITGB1
GO.0009966	regulation of signal transduction	7	BDNF, CD44, CD8A, ICAM1, IL15, IL8, ITGB1
GO.0071356	cellular response to tumor necrosis factor	3	BDNF, ICAM1, IL8
GO.0033627	cell adhesion mediated by integrin	2	ICAM1, ITGB1
GO.0045321	leukocyte activation	4	ICAM1, IL15, IL8, ITGB1
GO.0060548	negative regulation of cell death	5	BDNF, CD44, ICAM1, ITGB1, NTF4
GO.0009605	response to external stimulus	6	BDNF, CD8A, ICAM1, IL15, IL8, ITGB1
GO.0048584	positive regulation of response to stimulus	6	BDNF, CD44, CD8A, ICAM1, IL15, IL8
GO.0002456	T cell-mediated immunity	2	CD8A, ICAM1
GO.0007166	cell surface receptor signaling pathway	6	BDNF, CD274, CD8A, ICAM1, IL8, ITGB1
GO.0034112	positive regulation of homotypic cell-cell adhesion	3	CD274, CD44, IL15
GO.0050731	positive regulation of peptidyl-tyrosine phosphorylation	3	CD44, ICAM1, IL15
GO.1903039	positive regulation of leukocyte cell-cell adhesion	3	CD274, CD44, IL15
GO.0006955	immune response	5	CD274, CD44, ICAM1, IL15, IL8
GO.0009967	positive regulation of signal transduction	5	BDNF, CD44, CD8A, ICAM1, IL15
GO.0030098	lymphocyte differentiation	3	CD8A, IL15, ITGB1
GO.0030212	hyaluronan metabolic process	2	CD44, IL15
GO.0048675	axon extension	2	BDNF, ITGB1
GO.0051240	positive regulation of multicellular organismal process	5	BDNF, CD274, ICAM1, IL15, IL8
GO.1902531	regulation of intracellular signal transduction	5	CD44, CD8A, ICAM1, IL15, ITGB1
GO.0006952	defense response	5	BDNF, CD8A, IL15, IL8, ITGB1
GO.0070486	leukocyte aggregation	3	CD44, ICAM1, IL15
GO.0001934	positive regulation of protein phosphorylation	4	BDNF, CD44, ICAM1, IL15
GO.0002684	positive regulation of immune system process	4	CD274, ICAM1, IL15, IL8
GO.0003008	system process	5	BDNF, ICAM1, IL15, ITGB1, NTF4
GO.0033993	response to lipid	4	BDNF, ICAM1, IL15, IL8
GO.0048513	organ development	6	BDNF, CD8A, ICAM1, IL15, IL8, ITGB1
GO.0050776	regulation of immune response	4	CD8A, ICAM1, IL15, ITGB1
GO.0071347	cellular response to interleukin-1	2	ICAM1, IL8
GO.1901701	cellular response to the oxygen-containing compound	4	BDNF, ICAM1, IL15, IL8
GO.1902533	positive regulation of intracellular signal transduction	4	CD44, CD8A, ICAM1, IL15

After antibody microarray, highly enriched proteins function and their interactions with other proteins was analyzed to identify impacted biological processes using the STRING database, version 10.5 (Available online: http://string-db.org). (GO = gene ontology).

## Supplementary Materials

Supplementary materials can be found at http://www.mdpi.com/1422-0067/19/2/464/s1.

## Abbreviations

BDNF	Brain-derived neurotrophic factor
CD	Cluster of differentiation
CD8A	T-cell surface glycoprotein CD8 α chain
CD98	4F2 cell-surface antigen heavy chain
CM	Conditioned media
ECM	Extracellular matrix
EMT	Epithelial-to-mesenchymal transition
ERK	Extracellular signal-regulated kinase
FAK	Focal adhesion kinase
hTCEpi	Telomerase-immortalized human corneal epithelial cell line
ICAM1	Intercellular adhesion molecule 1
IFNA1	Interferon-α1
ITGB1	Integrin β1
IL-8	Interleukin-8
IL-15	Interleukin-15
IL-37	Interleukin-37
LIMMA	linear models for microarray data
MAPK	Mitogen-activated protein kinases
NTF4	Neurotrophin-4
PECA1	Platelet endothelial cell adhesion molecule
PD1L1	Programmed cell death 1 ligand 1
SFs	Stromal fibroblasts
SFCM	Stromal fibroblasts conditioned media
SP	Substance P
VASP	Vasodilator-stimulated phosphoprotein
ZEB1	Zinc finger E-box binding homeobox 1
