# Supplementary material for: Role of Corneal Stromal Cells on Epithelial Cell Function during Wound Healing
Source: Int J Mol Sci. 2018 Feb 4;19(2):464. doi: 10.3390/ijms19020464 (PMC5855686; doi:10.3390/ijms19020464)
Supplement: Supplementary file 1 [file ijms-19-00464-s001.pdf]

Suppl. information:

Target list of the molecules analysed during antibody microarray:

CD Marker	Gene name	CD Marker	Gene name	Chemokine / Cytokine	Gene name	Additional proteins	Gene name
CD1a	LEU6	CD50	ICAM3	GM-CSF	CSF2	ANCA	PRTN3
CD2	SRBC	CD52	HE5	IFN alpha	IFNA1	BDNF	BDNF
CD3	T3G	CD53	TSPAN25	IFN gamma	IFNG	Cox1	PTGS1
CD4	LEU3	CD54	ICAM1	IL-1alpha	IL1A	CYTL	CYTL1
CD5	LEU1	CD55	DAF	IL-1beta	IL1B	IgE	FCER1A
CD6	TP120	CD56	NCAM1	IL-2	IL2	HLA I	
CD7	LEU9	CD57	B3GAT1	IL-4	IL4	HLA-ABC	
CD8	MAL	CD58	LFA3	IL-6	IL6	HLA-DP	HLA-DPB1
CD9	MIC3	CD59	MIC11	IL-7	IL7	HLA-DQ	
CD10	MME	CD61	ITGB3	IL-8	CXCL8	HLA-DR	
CD11a	ITGAL	CD62L	SELL	IL-10	IL10	MPO	MPO
CD11b	ITGAM	CD62p	SELP	IL-12B	IL12B	NT-4	NTF4
CD11c	ITGAX	CD63	TSPAN30	IL-13	IL13	NTAL	LAT2
CD13	ANPEP	CD66a	CEACAM1	IL-15	IL15	pan HLA-class II	
CD14		CD66b	CEACAM8	IL-16	IL16	p53	TP53
CD15	FUT4	CD66c	CEACAM6	IL-18	IL18	p72Syk	SYK
CD16	FCGR3A	CD66d	CEACAM3	IL-37	IL37	TRYG1	TPSG1
CD17		CD66e	CEACAM5	LIF	LIF	TSLPR	CRLF2
CD18	ITGB2	CD69	CLEC2C	TNF alpha	TNF	tTG	TGM2
CD19	LEU12	CD70	TNFSF7	TSLP	TSLP	VEGF	VEGFA
CD20	MS4A1	CD71	TFRC				
CD21	CR2	CD72	Lyb-2	Eotaxin-1	CCL11		
CD22	SIGLEC2	CD79a	MB1	MIP-1 alpha	CCL3		
CD23	FCER2	CD80	LAB7	RANTES	CCL5		
CD24		CD86	LAB72	MCP-2	CCL8		
CD25	IL2RA	CD95	FAS	MCP-3	CCL7		
CD27	TNFRSF7	CD97		MIP-4	CCL18		
CD28	TP44	CD98	SLC3A2				
CD29	ITGB1	CD99	MIC2				
CD30	TNFRSF8	CD105	ENG				
CD31	PECAM1	CD106	VCAM1				
CD33	SIGLEC3	CD116	CSF2RA				
CD34		CD117	KIT				
CD35	CR1	CD123	IL3RA				
CD36	GP3B	CD131	CSF2RB				
CD37	TSPAN26	CD137	TNFRSF9				
CD38	ADPRC 1	CD139					
CD40	TNFRSF5	CD147	BSG				
CD41	ITGA2B	CD162	SELPLG				
CD42b	GP1BA	CD177	NB1				
CD43	SPN	CD222	IGF2R				
CD44	MDU2	CD223	LAG3				
CD45	PTPRC	CD230	PRNP				
CD46	MCP	CD235a	GYPA				
CD47	MER6	CD235b	GYPB				

CD48	BCM1	CD253	TNFSF10
CD49d	ITGA4	CD274	PDL1
		CD279	PD1

Suppl. Fig. 1

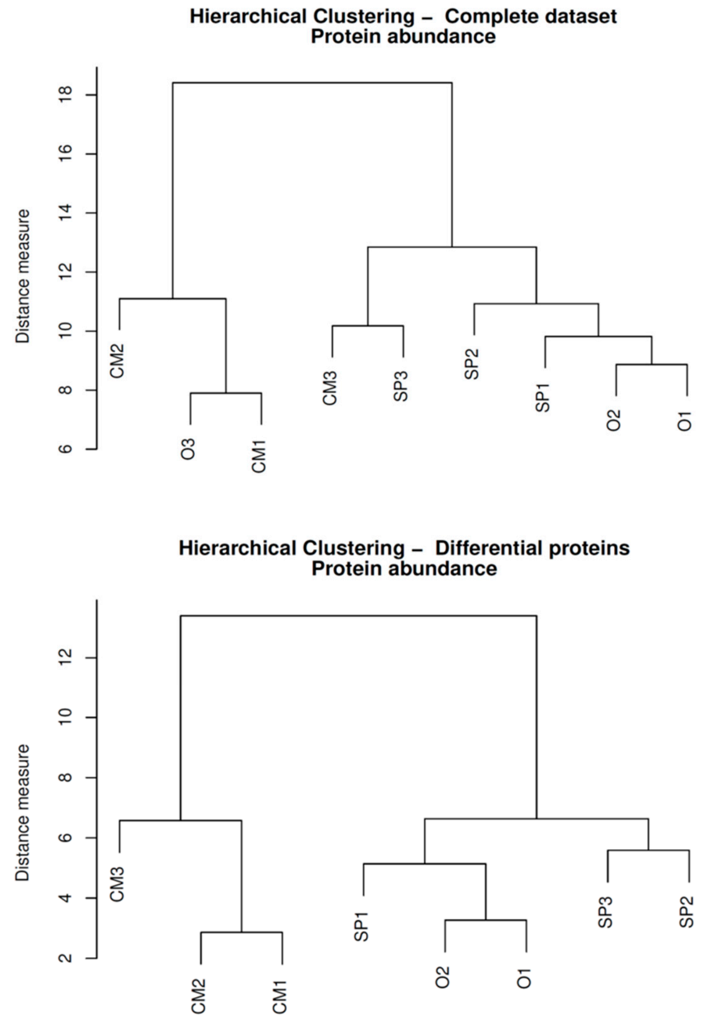

Suppl. Fig.1: Hierarchical clustering of the protein extracts based on the complete array data and differential abundance. O1, O2 and O3 represent control sample group. SP1, SP2 and SP3 represent substance P treated sample group. CM1, CM2 and CM3 represent keratocyte conditioned media (KSFM) treated sample group. The cluster analysis for the protein signal data of the complete data set reveals two main clusters. The samples from

group SP and O are clustering to the right whereas the CM sample group is located to the left. Sample O3 is classified as an outlier and filtered from the following analysis. A further cluster analysis was performed with only the differential data set which also revealed two main clusters. The cluster on the right contain samples from the O and SP group and on the left consists only of the CM sample group that was clearly separated from the other two groups.

Suppl. Fig. 2

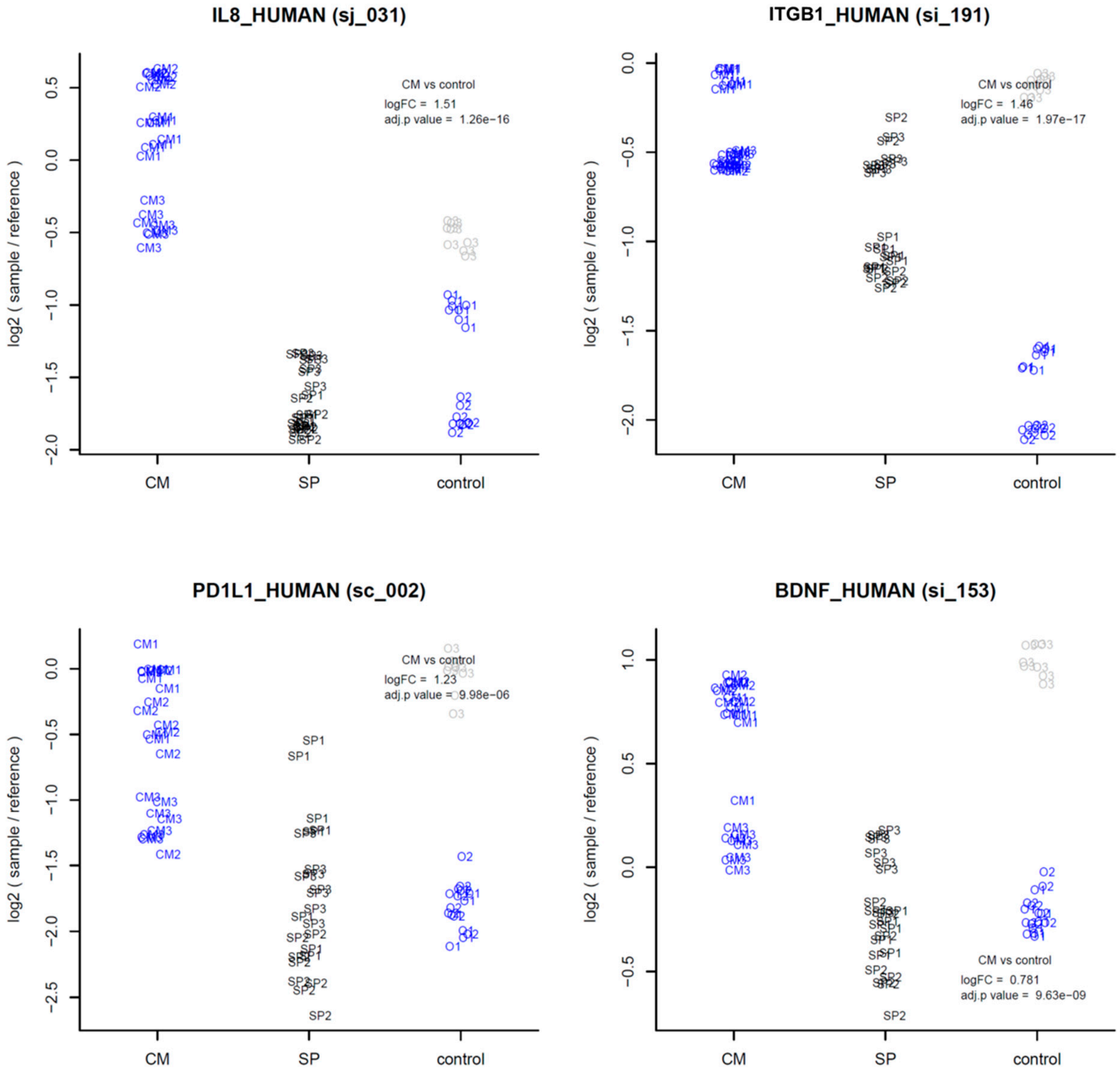

Suppl. Fig.2: Individual array values for a set of differentially expressed proteins. Each sample is measured by four replicate spots per array. Data are shown for proteins ITGB1 (upregulated after SP and KCM treatment), as well as IL8, PD1L1 and BDNF (upregulated after KCM treatment). The filtered sample O3 was also presented in the plots.

Suppl. Fig. 3

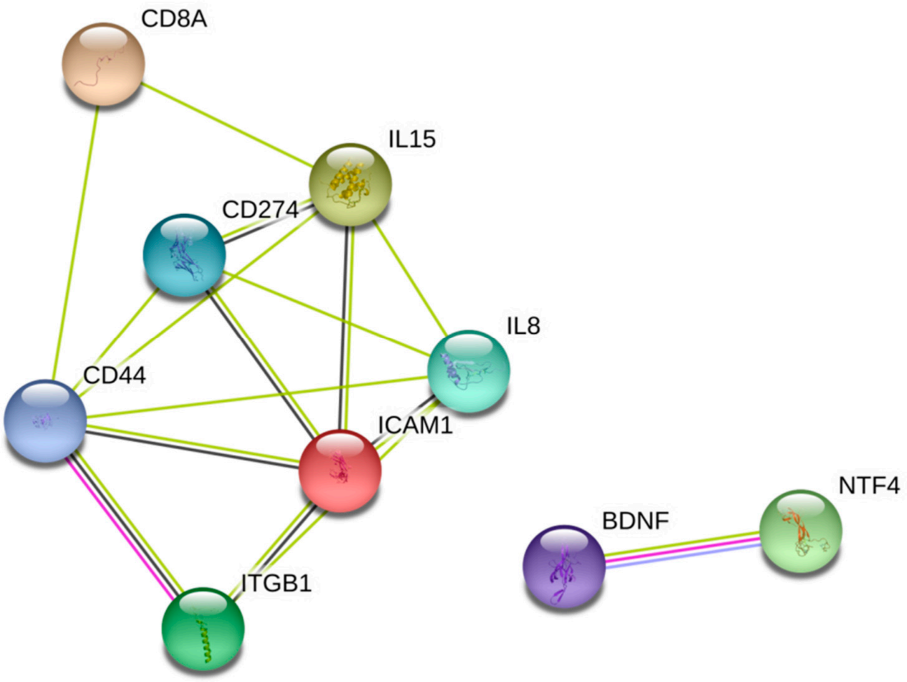

Suppl. Figure 3. Protein-protein interaction network obtained by STRING software (p-value = 6.37e-08). Differentially expressed abundant proteins, after 24-hour stimulation

of hTCEpi cells with SFCM, were identified by antibody microarray and their interaction analysis was performed using *version 10.5 of* the STRING database (http://string-db.org). Colored lines between proteins indicate different kinds of interaction evidence. Several direct, as well as indirect interactions of the differential proteins, were observed. In this network, nodes represent proteins and edges represent protein-protein associations. With 9 nodes and 13 edges, the represented interaction network displays more biologically connected protein interactions among themselves as well as a cluster.
